# The barriers and enablers of older person health assessments in Australian primary care: clinician and patient perspectives

**DOI:** 10.3399/BJGPO.2023.0091

**Published:** 2023-10-18

**Authors:** Nagalaxmi Iyengar, Eleanor Mitchell

**Affiliations:** 1 Department of General Practice, School of Public Health and Preventive Medicine Faculty of Medicine, Nursing and Health Sciences, Monash University, Melbourne, Australia; 2 Monash Rural Health, Monash University, Melbourne, Australia

**Keywords:** Patient perspectives, Care of the elderly, Qualitative research, Primary healthcare, General practice

## Abstract

**Background:**

Health assessments (HAs) were introduced for at-risk patients, including older people, to have their health comprehensively monitored by their GP, to assess specific areas of health, such as risk factors for chronic disease and psychosocial problems, which may be overlooked in shorter consultations. Two forms of older person HAs are available for GPs to perform annually, HAs for non-Indigenous older Australians aged >75 (75+ HA) and for Aboriginal and Torres Strait Islander Australians aged >55 years (55+ ATSIHA).

**Aim:**

The present study aimed to explore the perspectives of older Australians undertaking HA (both 75+HA and 55+ ATSIHA) and clinician perspectives (GPs and practice nurses [PNs]) to enhance the items covered within the HA and develop targeted education resources to improve uptake of HAs.

**Study & design:**

A qualitative study design incorporating semi-structured interviews and narrative inquiry was performed, inviting patients who have undergone HAs (75+HA and 55+ ATSIHAs) across two metropolitan general practice clinics. Clinicians who completed the HAs were also invited to participate in this study.

**Method:**

A total of 15 clinicians (11 GPs and 4 PNs) and 15 patients participated in this study. Thematic analysis was used to identify barriers and enablers of HAs.

**Results:**

Common barriers to both patients and clinicians include time, language, lack of relevance, and fear of the unknown. Identification of risk factors and the opportunity to discuss topics not covered in shorter consults were common enablers for both patients and clinicians.

**Conclusion:**

Four major patient barriers identified in this study include communication, accessibility, lack of engagement, and lack of patient preparation. The comprehensive nature of HAs was a major enabler for both clinicians and patients.

## How this fits in

Older person health assessments (HAs) are associated with increased longevity and improved patient satisfaction. An understanding of barriers and enablers of HAs is essential to improve HA uptake.

## Introduction

HAs were introduced in Australia in 1999 as a Medicare Benefits schedule (MBS) Item available for patients to have their health comprehensively monitored by their GP, underpinning the foundation of general practice.^1^ A vast majority of well-resourced nations including the UK,^2–4^ Canada, The Netherlands,^5^ and New Zealand have all employed HAs as a useful screening tool to identify risk factors in vulnerable patients, optimise patient care, and its implementation has been associated with improved longevity. Therefore, as average life expectancy continues to rise globally, an understanding of patient and clinician perspectives of the barriers and enablers of HAs are relevant across primary care and is transferable and translatable across the developed world.

In Australia, two forms of older person HAs are available for GPs to perform annually, HAs for non-Indigenous older Australians aged >75 (75+ HA, MBS item numbers 701, 703, 705, 707) and for Aboriginal and Torres Strait Australians aged >55 years (55+ ATSIHA, MBS item number 715).^1^ Specifically, HAs across developed nations are similar and require GPs, with the optional aid of a practice nurse (PN), to assess various parameters including a complete physical examination, mini-mental state examination, emotional and social well-being, need for home supports, frailty, home medication review, and an opportunity to practice preventative medicine, such as immunisation review and falls risk assessments.^6^ HAs also provide an opportunity to discuss difficult topics not otherwise discussed in shorter consultations, such as end-of-life care, appointing a medical power of attorney, palliative care, and referral to aged care services if indicated. These important topics are not routinely discussed in shorter consultations due to the time constraints and lack of contextual relevance to patients in day-to-day mainstream general practice.

Previous studies have shown that the 75+ HA uptake has substantially improved over the years, although huge variation in state and sex has been noted.^7^ Clinicians’ attitudes were generally positive regarding the usefulness of performing older person HAs, however practitioner time restrictions and patient refusal were identified as the predominant barriers to delivery.^8^ Healthcare professionals (GPs and PNs) also requested reintroducing HAs at a patient’s home, which was removed as an item in the Medicare Benefits schedule in 2010.^9^

Data to date suggest that Aboriginal and Torres Strait Islander people were significantly less likely to have HAs than the rest of the community.^10^ Although the uptake of ATSIHAs has been increasing, there is a high degree of regional variation in delivery and many Sydney-based health professionals interviewed by Schütze and colleagues^11^ still unaware of the ATSIHA. It has also been recently established that delivery of 75+ HAs to people >75 years was associated with a 5% lower risk of mortality when compared with individuals who had not received a 75+HA.^12^ Earlier research demonstrated HAs were associated with improvements in quality of life and patient satisfaction.^13–16^ While much of the published data have assessed clinician views on the usefulness of HAs, there is limited data on the patient perspective, the ultimate health beneficiaries.

The objective of the present study was twofold: 1) to explore the perspectives of older Australian patients regarding their experiences of undertaking HAs (75+ HA and 55+ ATSIHA); 2) to explore clinician perspectives (including GPs and PNs) of the barriers and enablers to conducting older person HAs.

The older person HAs are designed to fulfil a role in identifying and addressing chronic health problems in older patients that may otherwise have been overlooked at standard GP consultations. While there is limited knowledge available on the health benefits of HAs, a better understanding of the patient experiences, attitudes, and perspectives towards these assessments would be important in evidencing the role HAs in healthcare. Thus, exploring the patient perspective and clinician perspectives would assist in enhancing the items covered within the HA, as well as assist in developing targeted education resources to improve uptake of HAs. This information could be utilised in informing GPs and policymakers, with the aim of increasing the uptake of HAs and improving HAs overall.

## Method

### Study design and setting

This project was granted ethics approval from the Monash University Human Research Ethics Committee. To obtain a sufficient sample size, only two general practices (at which NI practised clinically, one inner metropolitan and one outer metropolitan) were recruited by purposeful sampling; clinicians (GPs and PNs) from both agreed to participate in this study. Patients were not known to NI prior to this study. Patients who had recently completed an HA within the last 12 months were invited by a PN (via phone or in person) to participate in this study, and included those who had the mental capacity to undertake an interview. Carers were also invited to attend the interview. Clinicians included GPs and PNs who routinely assist GPs in their HAs. Within these clinics HAs were conducted either entirely by the GP or in combination with the PN. A phone interpreter service was available to clinicians if required. Participants were recruited until data saturation was obtained. Patients were reimbursed with a $50 gift voucher for their participation. Participating GPs were reimbursed with a $250 gift voucher and PNs were reimbursed with a $50 gift voucher.

This study adopted an inductive phenomenological approach to understand the personal experiences of patients and clinicians involved in HAs to gain a rich understanding of the barriers and enablers of HAs. The authors used this to generate themes to inform strategies as to how HAs can be improved, with the ultimate aims of supporting increased uptake and better serving the health needs of older Australians.

Semi-structured interviews were conducted with consenting participants by NI. Patient interviews were conducted over the telephone, while clinician interviews were conducted face to face, online, or over the telephone. Open-ended questions allowed opportunity for further discussion. All interviews of patients and clinicians were de-identified, and both telephone and face-to-face interviews were recorded with consent on the online Zoom platform. Interviews included some demographic questions and a range of targeted questions to understand the barriers and enablers of HAs. The specific questions used to initiate conversation can be found in Supplementary Figure 1. All interviews were de-identified and stored securely.

Audio recordings of interviews were transcribed verbatim by Otter.ai and were analysed using thematic analysis guided by Braun and Clarke’s six-step method.^17^ Interviews were conducted by NI (academic GP registrar, Monash University) who constructed the initial coding framework, identified themes and sub themes, and performed context analysis. All interview transcripts and recordings were also independently reviewed and analysed by EM (an experienced primary care researcher), with a high standard of intercoder reliability between the two researchers.

## Results

In this study, the authors conducted semi-structured interviews with patients and clinicians to understand the barriers and enablers of HAs. A total of 15 patients and 15 clinicians across two metropolitan general practices participated in this study. Clinician interviews ranged between 6–25 minutes in duration (average duration 14.2 minutes) and patient interviews ranged between 5–21 minutes in duration (average duration 10.1 minutes).

Clinicians included 11 GPs and 4 PNs. GPs interviewed were at least 4 years post Royal Australian College of General Practitioners fellowship. Of the included GPS, *n* = 4/11 were from the inner metropolitan clinic, while *n* = 7/11 were from the outer metropolitan clinic. PNs included registered nurses from an inner metropolitan clinic (*n* = 1) and *n* = 3 from the outer metropolitan clinic. All GPs undertook HAs together with the aid of a PN who initially reviewed the patient and conducted the initial assessment. Two GPs stated that on occasion they conducted the entire HA themselves due to linguistic and cultural needs of the patient. Aside from language, other clinician barriers included time, education and cultural barriers, lack of patient understanding, and cognitive impairment.

Of the 15 patients included, 14 underwent 75+HA and the average age of the patient was 78.9 years. One patient, who was 62 years of age, completed the 55+ ATSIHA. Five patients were from the inner metropolitan clinic, while *n* = 10/15 patients were from the outer metropolitan clinic. Overall, all patients (*n* = 15/15) agreed that HAs were a useful tool to provide a comprehensive assessment of their health. No obvious differences were noted in the response of patients between the inner metropolitan and outer metropolitan clinics. Four main themes were identified as patient barriers for HAs: communication (*n* = 2/15); accessibility (*n* = 2/15); lack of engagement (*n* = 2/15); and lack of patient preparation (*n* = 2/15). One patient also cited time as a barrier for engaging in HAs.

Four major clinician barriers were noted: language (*n* = 7/15), cultural, and educational barriers (*n* = 2/15); time (*n* = 15/15), and funding (*n* = 3/15); impact on the doctor–patient relationship (*n* = 3/15); and patient cognition (*n* = 4/15). In addition, a number of sub-themes were identified within the major themes presented for both patients and clinicians.

### Patient barriers associated with older person HAs

Thematic analysis of patient barriers for older person HAs:

#### i) Communication

Many patients reported difficulty undertaking HAs due to language barriers. Although patients were aware that an interpreter service could be offered, patients preferred to attend with a family member to overcome the language barrier so they could assist as an interpreter, or felt the need to see a doctor who spoke the same language to undertake a HA:

'*Language is a problem*.' (Outer metropolitan, patient 2)

'*I had no idea what they were actually asking*.' (Outer metropolitan, patient 3)

#### ii) Accessibility

Difficulty attending the clinic in person was cited as a barrier to attending HAs. Patients often had to rely on family to escort them to the clinic. Two of the 15 patients who underwent HAs in this study were accompanied by their daughters to undertake a HA:

*'It’s not so easy for them to get to places where they can't drive or they don't drive or, you know, they're away from public transport*.' (Outer metropolitan, patient 4)

One patient was also surprised to learn that funding to conduct HAs at home was no longer possible and she noted that her mother had previously undertaken a HA at home:

'*That’s ridiculous. She became immobile and ended up needing a wheelchair. She had to be assessed at home.'* (Outer metropolitan, patient 5)

#### iii) Lack of engagement

Some patients found the HA was not relevant to their current level of health and fitness. At least *n* = 2/15 patients were still in active employment and were offended that the HA questions had the general assumption that people lived at home and didn’t work:

'*They assumed that I lived at home and didn't work.'* (Inner metropolitan, patient 1)

The manner in which the HA questions were posed were also perceived as patronising to some patients:

'*I didn't like the fact that some of the questions were fairly juvenile in terms of what your name was, what your address was, particularly after you've visited the clinic many times. And also, what day of the week it was. I mean, I used to be a manager … I used to be a tax agent. I worked very hard and very long, and … I believe I have a terrific brain. My body’s not so good. But my brain is probably a few years in front of my body. And questions like that just seem to be a little bit silly.'* (Inner metropolitan, patient 1)

#### iv) Lack of patient preparation

Many patients did not understand or receive education regarding the purpose and need to undertake a HA. Some patients felt it was thrust on them by their doctors, whom they had known for many years, and did not understand the medical or personal benefits of HAs for patients:

'*It was really put on; it wasn't something I'd chosen to do. I thought it was just part of the system.'* (Inner metropolitan, patient 2)

Two patients viewed HAs as a tool that would primarily benefit clinicians by providing them with increased remuneration. One patient assumed that the HA provided the same benefit as a chronic disease management plan and did not provide any further advantage for patients, but saw HAs as an opportunity for increased remuneration for clinicians:

'*The medical profession, maybe they try to separate the two to get fees from the government?*' (Inner metropolitan, patient 2)

One patient also commented that time was a barrier to HA uptake due to needing to be seen by a nurse followed by a doctor for the completion of a HA, and expressed frustration of the lengthy waiting times that are common in general practice:

'*They're* [doctors] *always late and that’s a disgrace because my time is more valuable than the doctor’s time.'* (Inner metropolitan, patient 2)

### Clinician barriers associated with older person HAs

#### i) Language, cultural, and educational barriers

The following themes were amalgamated due to an interrelationship between these factors evidenced by the need for an interpreter to overcome language barriers; a preference for alignment of cultural nuances with patients preferring to consult with a doctor of similar cultural background; and perceived feelings of fear or shame among patients due to presumed language insufficiency, cultural differences, or lack of education due to circumstance. Clinicians also perceived language to be a barrier to HAs because of the need to involve an interpreter (professional or family member), which made the assessment even more time-consuming:

*'If I'm using an interpreter, it can take triple the amount of time.'* (Outer metropolitan, GP1)

Overall, patients preferred to see a GP who spoke the same language and understood the subtle cultural nuances of a doctor–patient relationship. This meant the GPs of a similar cultural background to the patient often undertook the HA entirely themselves, without the assistance of the PN:

*'I have a lot of patients* [from the same cultural background as myself] *who don’t speak any English. So, the health assessment, the whole thing would have to be done by me. So that takes a big chunk out of the session.'* (Outer metropolitan, GP1)

Cultural barriers included societal perception of the role of the doctor as the healer and the rescuer, and the difficulty identifying risk factors and practising preventative medicine due to perceived cultural norms:

*'My patients don't want to have any assessment. They just want what’s necessary, then they are fine. Also, they don’t want to be put into a nursing home. Well, you know, it’s a cultural thing, I suppose. Yeah. As well as the bit of paranoia that comes with whatever it is.'* (Outer metropolitan, GP1)

An experienced primary care registered nurse discussed limited schooling as a HA barrier, which may also contribute to a general disinterest in HAs and fear of undertaking a HA due to associated feelings of fear and shame as it belittled their capacity:

'*Admitting that they never attended school … the mathematical questions are too hard*.' (Outer metropolitan, PN1)

#### ii) Time and funding

Experienced clinicians were in agreement that further background could be gleaned by conducting the HA in the patient's home but also discussed some of the associated challenges:

'*Look, there’s no doubt in my belief, it’s ... it would be much more beneficial to do it in their own* [home] *probably to some extent, like doing house calls … I think, I think when you see people in the clinic room, you don't really get a true understanding of how the domestic situation is. So, you know, how they are negotiating rooms, getting access from beds, to toilets, getting out of chairs, the condition of how they're living. I mean, all these sorts of things you can't get from the verbal interview. So, I would certainly prefer to do it in a face-to-face setting in the home.'* (Outer metropolitan, GP2)

'*But from a practical point of view, that’s not always possible because of the time constraints. And also, the other problem is that, you know, for some of these elderly* [older] *patients, they require either a carer or a family member to be present. And there’s the difficulty of coordinating times.'* (Outer metropolitan, GP2)

#### iii) Impact on the doctor–patient relationship

Some patients felt that doctors had a hidden agenda behind asking the patient to undertake the HA, and feared losing their independence. Many clinicians also felt that patients in general did not enjoy the experience of sharing their personal experience in the HA and that it negatively impacted the doctor–patient relationship:

'*They feel like you've exposed them to somebody who’s asked them these really harsh questions.'* (Outer metropolitan, GP3)

#### iv) Patient cognition

Patient cognition was identified as a barrier to HAs as it was found to affect the level of patient engagement and interest, as they did not want to be confronted with evidence of physical or mental deterioration in capacity:

'*People don't want to know they're getting older, and they're not able to function as they used to be, you know, either mentally or physically.'* (Outer metropolitan, GP3)

'*If you do have a patient that’s not wanting to come in,* [it’s] *because they realise they are needing help.'* (Outer metropolitan, GP3)

A summary of the major patient and clinician barriers of heath assessments identified can be reviewed in Supplementary Table S1.

### Patient and clinician enablers of HAs

Overall, four major themes were identified as patient enablers for HA uptake. Patients unanimously agreed that HAs raised confidence in current health and valued the opportunity to cover health concerns that do not always arise in shorter consults. It was also seen as an opportunity to establish common health goals between the clinician and the patient, and the patient appreciated the comprehensive nature of the HA. Four clinician themes were also identified: clinicians also valued the comprehensive information gleaned from HA evaluation, formalising the review provided an opportunity to discuss difficult topics that appear out-of-context to the patient in mainstream general practice. Clinicians also found it rewarding to practice preventative medicine, to review, and update the patient's file and some also suggested that an enabler of HAs was the opportunity for further remuneration for GPs, compared to a standard consultation fee.

### Thematic analysis of patient HA enablers

#### i) Raises confidence in current health

Overall, patients unanimously agreed that HAs raised confidence in their current health status as it included detailed questioning of broad aspects of their physical and mental well-being and their supports:

*'If anything didn't seem quite right. I would get told about it*.' (Inner metropolitan, patient 2)

Patients also viewed HAs as an opportunity to discuss any of their own concerns in a safe and respected environment:

'*It just takes a bit of pressure off yourself … that you're not going nutty?*' (Outer metropolitan, patient 4)

#### ii) Opportunity to cover health concerns that do not always arise in shorter consults

The broad nature of the HA coverage allowed the patients opportunity to discuss issues that they would not normally require a medical consultation for, such as home support:

'*It was smaller, more relaxed, and I could talk about little things. And that was great. Because I tend to hold a lot of things back a bit … by talking to somebody professional it’s released, gives you* [some] *life*.' (Outer metropolitan, patient 4)

#### iii) Ensures that everyone is on the same page

Establishing common goals was a key patient enabler of HAs. Patients found it empowering to witness having their personal information, strengths, and limitations documented on file:

'*I've got everything on record, I suppose.'* (Inner metropolitan, patient 2)

#### iv) The comprehensive nature of the HA

Patients valued the comprehensive nature of the HA as it allowed the patient to have a good baseline summary of their health documented on record. Patients understood that this could be used as a baseline screening tool to monitor any subtle deteriorations in their capacity or well-being:

*'It covers such a broad range of things. You talk about things that you might not otherwise talk about, you know, with a doctor in a short consultation.'* (Inner metropolitan, patient 3)

*'Everything done in one hit.' (Inner metropolitan, patient 4*)

### Clinician enablers

#### i) Comprehensive coverage of the HA

Clinicians also appreciated that HAs allowed a comprehensive assessment of the biopsychosocial needs of the patient. It allowed the identification of risk factors, such as risk of falls, and ensured that care was up-to-date:

*'You feel like you've genuinely understood the patient.'* (Outer metropolitan, GP4)

'*It would give a comprehensive assessment of their whole life, I suppose and whether they've missed anything, for example, immunisations.'* (Outer metropolitan, GP4)

#### ii) Opportunity to discuss difficult topics

The time pressures and day-to-day demands of general practice limit the opportunity to discuss important topics that may seem out of context to the patient, and HAs are an ideal opportunity to raise these issues in a formalised manner. These include difficult topics such as end-of-life care and appointing a power of attorney:

*'I think HAs are great because we go into areas that normally don't come up, they're not always on the patient’s agenda.' (Outer metropolitan, GP4*)

#### iii) Enabled the practice of preventive medicine

As patient files were routinely updated during the HAs, it was also noted to be a good opportunity to practice prevention of deterioration and disease.

'*We identify their needs, and then we can guide them for necessary available services*.' (Inner metropolitan, GP1)

*'Because it’s so comprehensive, you end up reviewing things like, hearing, how they function, and safety, you know, falls risk so and can prevent things from happening. So, you know, you'd rather see the patient and talk about things that they can do to reduce falls than see them after a fall*.' (Outer metropolitan, GP4)

'*Well, I feel like you know, at least once a year, I get a good idea of how my patients are truly coping, it’s identifying problems that might arise before* [they arise], *it’s prevention medicine at its best, basically.'* (Outer metropolitan, GP4)

#### iv) Opportunity for increased remuneration for GPs

Clinicians (*n* = 2/15) reported that the financial rebate associated with the annual HA was a good incentive and motivator for GPs to undertake annual HA to better understand their patients:

'*If I was completely 100% honest, what I would say is that health assessments are these little financial rewards that Medicare allows us to claim in order to do what we should be doing regularly anyway.'* (Inner metropolitan, GP2)

### Recommendations to improve HAs

Patient recommendations to improve HA uptake and relevance included specific discussion on pain, sleep, grief, and bereavement, and they welcomed the opportunity to discuss cancer screening. Patients also expressed the need for dental review as an important component of older person HA. Clinician suggestions included assessing fitness to drive and review of patient financial well-being as part of HAs.

A summary of the major patient and clinician enablers of HAs can be found in supplementary Table 2.

## Discussion

### Summary

The present study evaluated the barriers and enablers of older person HAs across two Australian general practices by analysing in depth responses to semi-structured interviews of clinicians and patients. Common barriers to both patients and clinicians include lack of time, language barriers, lack of relevance, fear of the unknown and fear of the potential negative consequences on the doctor–patient relationship due to the comprehensive nature of the HAs. Identification of risk factors and the opportunity to discuss difficult topics, such as end-of-life care that are not covered in shorter consults, were common enablers for both patients and clinicians.

Clinicians (GPs and PNs) also recommended that it would be useful to undertake the HA in the patient’s home setting to better appreciate the home living situation to identify risk factors. Funding to undertake HAs in the home setting was removed in Australia in 2010, and this study's findings suggest that this may need reviewing to improve uptake of HAs in patients that have limited accessibility or are not mobile.

### Strengths and limitations

There are few accounts to date on patient perspectives of HAs that include the views of an Aboriginal elder, as this study does. The current uptake of HAs by older Australians is less than 40%, therefore the findings of the present study would assist in greater targeting to promote these within the clinical setting.^7^

A limitation of this study includes that only two clinics participated in this study from which GPs, PNs, and patients were recruited. While this may have led to selection bias, neither the clinicians nor the participants were involved in any aspect of the study design or execution. As well as including a broader cohort of patients, further research reviewing recent trends in 55+ ATSIHA uptake as well as focusing on Aboriginal perspectives of the barriers and enablers of older person HAs would also lead to improved HA uptake by allowing targeted promotion.

An addition source of potential bias is that only patients who had completed the HAs were invited to interview. Patients who had declined the assessments may have had very valuable views about the barriers to their attendance.

### Comparison with existing literature

Previous studies have identified HAs as a comprehensive tool to enable clinicians to identify cardiovascular issues and psychosocial problems.^7^ Strumberg^18^ demonstrated that HAs were useful to identify falls risk factors consistent with the idea of HAs enabling the practice and implementation of preventative medicine. Blakeman *et al* ^19^ have highlighted the identification of social problems through 75+ HA delivery. Ramisetty *et al* demonstrated that, when compared with standard GP consultations, 75+ HAs identify different types of new health problems, including elevated lipids, vitamin D deficiencies, eye and vision-related conditions, diabetes, and hearing conditions. Furthermore, targeted referrals and further management of previously identified problems occur at 75+ HA.^8^ Taken together, the findings of these studies are consistent with the theme generated from the present study that suggested that HAs provide a comprehensive overview of the biopsychosocial needs of the older patient.

To promote patient engagement, improve HA uptake, and overcome perceived barriers to the doctor–patient relationship, HAs can be normalised as a screening tool to patients to identify risk factors in healthy older patients in mainstream general practice. Many of the healthy older patients interviewed in the present study stated that attending annual health checks from the age of 75 was useful to provide a baseline in comparison to assess frailty over time, which cannot otherwise be gleaned in shorter consultations.

### Implications for research and practice

Distinguishing barriers and enablers at various levels related to patient characteristics, doctor–patient relationship, preparation for the HA encounter and process, organisational context, and the wider economic and policy context may also yield information to better understand correlations to promote targeted HA uptake. Only patients who had completed the HAs were invited to interview, and patients who had declined the assessments may have had very valuable views about the barriers to their attendance; this represents a useful direction for future studies to address specific patient barriers that limit HA uptake. The impact of COVID-19 on 75+ HA uptake may also be of interest to assess more recent trends.

Given that the Australian Government has spent over $135 million on older person HAs in 2019 alone to support its rising ageing population, it is important that the value of the 75+ HA continues to be well researched and monitored. Overall, the present study presented a comparative overview of the barriers and enablers of older person HAs in Australian primary care informing GPs and policymakers strategies to increase the uptake of HAs and their overall improvement.

## Data

The dataset relied on in this article is available from the corresponding author on reasonable request.
